# Supplementary material for: The phylogeny of desmostylians revisited: proposal of new clades based on robust phylogenetic hypotheses
Source: PeerJ. 2019 Oct 17;7:e7430. doi: 10.7717/peerj.7430 (PMC6800978; doi:10.7717/peerj.7430)
Supplement: File S1 [file peerj-07-7430-s001.docx]

**Character list**

**Teeth**

**General dental morphology**

1. Tooth column: normal (0), with thickened enamel (1) (after Inuzuka, 2005, character 26).
2. Dental root: relatively short (0), extremely elongated (1) (after Inuzuka, 2005, character 5).

**Canines**

1. Upper canine: present (0), absent (1) (after Beatty, 2009, character 26).
2. Lower canine tusk: circular in cross section (0), mediolaterally compressed (1) (after Beatty, 2009, character 27).
3. Lower canine root: not enlarged in diameter (0), enlarged in diameter to form a tusk (1) (after Barnes, 2013, character 17).
4. Lower canine crown: small (0), enlarged (tusk-like) (1) (after Clark, 1991, character 5).

**Premolars**

1. p1: present (0), absent (1) (after Clark, 1991, character 7).
2. p2: present (0), absent (1) (after Clark, 1991, character 8).
3. p2: pre-molariform, with a small cusp anterior to the main cusp and small talonid (0), with a main cusp and a talonid cusp only(1), caniniform (2)  (after Clark, 1991, character 10).
4. p2: single-rooted (0), double-rooted (1), with fused double roots (2) (after Clark, 1991, character 11).
5. p3: present (0), absent (1) (after Barnes, 2013, character 18).
6. p3: paraconid present (0), lost (1) (after Beatty, 2009, character 12).
7. p4: paraconid present (0), reduced to a vestige (1), absent (2) (after Barnes, 2013, character 19 and Beatty, 2009, character 12).
8. Roots of p3 and p4: single-rooted (0), double-rooted (1), with fused double roots (2) (after Clark, 1991, character 12).
9. p4 talonid: two posterior cusps, the hypoconid and entoconid, equal in height to the anterior two cusps, the protoconid and metaconid (0), the hypoconid and entoconid reduced in height (1) (after Barnes, 2013, character 20).

**Molar**

1. Hypoconulid shelf of M3: not broadened (0), transversely broad (1) (after Beatty, 2009, character 1).
2. Molar cusps: not swollen, separated (0), swollen and appressed (1) (after Beatty, 2009, character 8).
3. P4-M3 hypoconulid and entoconid: not enlarged (0), enlarged, especially entoconid (1) (after Beatty, 2009, character 9).
4. m2: without extra cuspid (0), with incipient extra cuspid between and labial to protoconid and hypoconid (1), with cuspid well developed (2) (after Beatty, 2009, character 10).
5. Extra cusps of molars: absent (0), incipient (1), large, approaching size of main cusps (2) (after Beatty, 2009, character 15).
6. Molars: brachydont (0), hypsodont, cusps columnar (1) (after Beatty, 2009, character 21).
7. Bony swelling medial to lower molars: absent (0), present (1) (after Beatty, 2009, character 24).
8. m3: with hypoconulid present (0), absent (1) (after Barnes, 2013, character 22).
9. m1: as long as, or longer than, p4 (0), smaller than p4 (1) (after Clark, 1991, character 15).
10. m2: without cusp labially between the protoconid and hypoconid (0), with cusp (1)  (after Clark, 1991, character 16).
11. Cusps on molar: asymmetrical and strongly tapered (0), columnar and gradually tapered with thick enamel (1)  (after Clark, 1991, character 18).
12. Cingulum on molariform teeth: forms distinct ridge (0), forms low swelling (1), absent (2) (after Clark, 1991, character 20).
13. Number of major cusps on M2: less than 6 (0), 6 or more (1)  (after Chiba et al., 2016, character 38).
14. Number of major cusps on M3: less than 7 (0), 7 or more (1) (after Chiba et al., 2016, character 39).

**Incisor**

1. Lower incisors: anteroposteriorly or obliquely aligned (0), transversely aligned (1) (after Beatty, 2009, character 4).
2. Number of lower incisors: three (0), two (1), one (2), zero (3) (after Beatty, 2009, character 16).
3. Shape of lower incisors: simple and small (0), flattened (1), conical and tusk-like (2) (after Beatty, 2009, character 22).
4. Pairs of upper incisors: zero (0), one (1), two (2), three (3) (after Beatty, 2009, character 23).
5. Incisors: with enamel (0), lacking enamel (1) (after Beatty, 2009, character 30).
6. i2 crown: medial and lateral margins parallel sided (0), expanded transversely to the extent that its lateral margin is curved laterally (1) (after Barnes, 2013, character 15).

**Skull**

1. Foramen within squamosal passing anterior from external auditory meatus: absent (0), present (1) (after Clark, 1991, character 21).
2. Passage anterior to external auditory meatus: connects with skull roof (0), does not connect (1) (after Clark, 1991, character 22).
3. Posterior part of premaxilla: just behind of alveolus of incisors and canine (0), elongate to much behind alveolus of incisors and canine (1) (modified after Clark, 1991, character 26).
4. External auditory meatus: low on skull, open ventrally (0), high on skull, closed ventrally (1) (after Beatty, 2009, character 2).
5. Paraoccipital process: not elongated (0), elongated (1) (after Beatty, 2009, character 5).
6. Foramen post-zygomaticus: absent (0), present (1) (after Beatty, 2009, character 6).
7. Premaxilla: does not contact the frontal (0), contacts the frontal (1) (after Beatty, 2009, character 18).
8. Sagittal crest: present (0), absent (1) (after Beatty, 2009, character 19).
9. Zygomatic process of the squamosal: not dorsoventrally broadened (0), broadened (1) (after Beatty, 2009, character 25).
10. Inter-premaxillary dorsal tuberosity: absence of a tuberosity anterior to the external nares on the dorsal surface of the suture between the premaxillary bones (0), presence of such a tuberosity (1) (after Beatty, 2009, character 34).
11. Anterior orbital groove: absent (0), present (1) (after Beatty, 2009, character 35).
12. Infraorbital foramen placement with respect to the orbit: in the same coronal plane with the orbit (0), in a coronal plane anterior to that of the orbit (1) (after Beatty, 2009, character 36).
13. Post-zygomatic foramen orientation with respect to the epitympanic sinus: foramen lies either directly anterior or antero-superior to the epitympanic sinus (0), foramen lies antero- inferior to the epitympanic sinus (1) (after Beatty, 2009, character 37).
14. Basioccipital bone: length is more than twice of the width of the foramen magnum (0), less than twice (1) (after Inuzuka, 2000, 2005, character 1).
15. Braincase expansion: less than half of the zygomatic width (0), more than half (1) (after Inuzuka, 2000, 2005, character 8).
16. Zygomatic process of squamosal: almost horizontal or slightly inclined to cranial side side (0), caudally inclined (1) (modified after Inuzuka, 2000, 2005, character 31).
17. Paroccipital process: normal (0), thickened (1) (after Inuzuka, 2000, 2005, character 32).
18. Median nuchal line: the crest not connected to superior nuchal line (0), the crest clearly connected to superior nuchal line (1) (after Inuzuka, 2000, 2005, character 34).
19. Skull: width/length (estimated) = about 40% and more (0), smaller than 40% (1) (after Inuzuka, 2000, 2005, character 37).
20. The position of nasal cavity: low (0), high (1) (after Inuzuka, 2000, 2005, character 38).
21. Zygomatic arch: not inclined at dorsal edge (0), medially inclined (1) (after Inuzuka, 2000, 2005, character 44).
22. Zygomatic arch: shorter than 1/3 of the skull or longer than 1/3 of skull length and position is the middle height of the skull (0), longer than 1/3 of skull length and position is the middle height of the skull (1) (after Inuzuka, 2000, 2005, character 45).
23. Dorsal narial opening: restricted to the anterior end of the snout (0), enlarged and extended posteriorly relative to orbit position (1) (after Barnes, 2013, character 3).
24. Nasal bones: the length is clearly longer than orbital width (0), almost the same or shorter than orbital width (1) (after Barnes, 2013, character 4).
25. Nasal bones: the maximum length of nasal bone is less than half width of the snout (0), the maximum length of nasal bone is more than half width of the snout (1) (after Barnes, 2013, character 5).
26. Dorsal surfaces of the supraorbital processes of the frontal: situated below the frontal (0), elevated dorsally above the frontal (after Barnes, 2013, character 6).
27. Supraorbital processes of the frontals: small and not projecting very far laterally (0), widened to the extent that the lateral margins of the postorbital processes project laterally beyond the jugal bones of the zygomatic arches (1) (after Barnes, 2013, character 7)．
28. Posterior end of the jugal bone: extends posteriorly to reach the anterolateral corner of the glenoid fossa (0), retracted anteriorly and does not reach the glenoid fossa (1) (after Barnes, 2013, character 9).

**Mandibular**

1. Upper margin of mandibular body: slight curved posterioly(0), sigmoid (1) (after Inuzuka, 2000, 2005, character 30).
2. Angle between the anterior and posterior margins of coronoid process: large (0), small (1) (after Inuzuka, 2000, 2005, character 24).
3. Coronoid crest (= anterior margin of ascending ramus) of the dentary: ascending vertically and curving posteriorly (0), curving anteriorly (1) (after Barnes, 2013, character 10).
4. Mandibular body of medial and lateral view: straight (0), ventrally flexed behind p2 (1) (after Inuzuka, 2000, 2005, character 23).
5. Mandibular symphysis: inclined anterodorsally (0), rotated anteroventrally to become approximately horizontal so that incisors and canines are directed anteriorly (1) (after Barnes, 2013, character 11).
6. Length of mandibular symphysis: less than 25% (0), about 30% (1), more than 40% (2) of the total length of the mandible (after Inuzuka, 2000, 2005, character 35).
7. Interalveolar margin of the mandible: not expanded laterally (0), laterally expanded (1) (after Inuzuka, 2000, 2005, character 43).
8. Diastema between Canie and cheek teeth: absent (0), present (1) (after Clark, 1991, character 6).
9. Diastema between p2 and p3: absent (0), present (1) (after Clark, 1991, character 9).

**Postcranial**

**Trunk**

1. Sternebrae: unpaired (0), paired (1) (after Beatty, 2009, character 32).
2. Bones of limbs: terrestrial type (=having medually cavity) (0), osteosclerosis (1), pachyosteosclerosis (2), cancellous (3) (after Beatty, 2009, character 20)
3. Bones of vertebrae (centrum): terrestrial type (=tight trabecular network but rather thin cortex) (0), osteosclerosis (1), pachyosteosclerosis (2), cancellous (3) (after Beatty, 2009, character 20).
4. Ribs: section is oval (0), flat (1) (modified after Inuzuka, 2000, 2005, character 36).
5. Ribs: strongly curved (0), almost straight (1) (newly added)
6. Thoracic vertebrae, number: 13 (0), 14 or 15 (1), 16 (2), 17 or more (3) (after Barnes, 2013, character 23).
7. Thoracic vertebra transverse processes: projecting laterally, directed away from the vertebral centra (0), inclined dorsolaterally relative to the centra (1) (after Barnes, 2013, character 24).
8. Lumbar vertebrae, number: 5-6 (0), 7 (1) (after Barnes, 2013, character 25).
9. Sacral vertebrae, number: five (0), reduced in number to four (1) (after Barnes, 2013, character 26).
10. Mesosterna: four pairs (0), three pairs (1) (after Barnes, 2013, character 27).
11. Centrum: the ring like shape epiphyseal line is absent (0), exists (1) (newly added)
12. Spinous process of thoracic vertebra: approximately straight (0), backwardly inclined (after Inuzuka, 2000, 2005, character 12).
13. Body size: less than 1.5m (0), between 1.5 to 2m (1), between 2m to 2.5m (2), more than 2.5m (3) (newly added).

**Forelimb**

1. Subscapular fossa: nearly flattened (0), clearly recessed (1) (after Inuzuka, 2000, 2005,

character 14).

1. Scapula, anterior border: nearly straight (0), slightly curved anteriorly (1), strongly curved anteriorly (2) (after Barnes, 2013, character 28).
2. Scapula, area of teres major muscle attachment on posterior border: concave and rugose (0), smooth, rounded, and convex (1) (after Barnes, 2013, character 29).
3. Humerus, proximal extension of greater tubercle: almost same level as the femoral head (0), extended above the femoral head (1) (newly added).
4. Humerus, width of greater tubercle: more than 1/2 of the width of the greater tubercle (0), less than 1/2 of the width of the greater tubercle (1) (newly added).
5. Humerus, lesser tubercule: prominent (0), indistinct (1). (newly added).
6. Humerus, intertubercular groove location: on cranial side (0), on medial side (1) (newly added).
7. Humerus, shape of intertubercular groove: deep, and wide (0), shallow and narrow (1), shallow and wide (2) (newly added).
8. Humerus, trochlea: enlarged or unchanged in diameter (0), decreased in diameter (1) (after Inuzuka, 2000, 2005, character 15).
9. Humeral crest: medially bent (0), the crest line is almost straight (1) (after Inuzuka, 2000, 2005, character 22).
10. Ulna, olecranon process: narrow both anteroposteriorly and transversely (0), widened anteroposteriorly and expanded medially (1) (after Barnes, 2013, character 30).
11. Ulna, olecranon process: relatively short, not lengthened proximally (0), olecranon process of the ulna in a proximal direction exists (1) (after Barnes, 2013, character 31).
12. Ulna, posterior border of diaphysis: nearly straight (0), bowed anteriorly, creating a concave posterior margin of the diaphysis (1) (after Barnes, 2013, character 32).
13. Radius and ulna: separated (0), fused (1) (newly added).
14. Third metacarpal base: not projected (0), proximally projected in the middle (1) (after Inuzuka, 2000, 2005, character 25).

**Hindlimb**

1. Capitate bone, orientation of distal articular facet: transverse (0), distal surface inclined medially (1) (after Barnes, 2013, character 34).
2. Femur, lesser trochanter: represented by only a muscle scar (0), forms a tubercle (1) (newly added).
3. Femoral shaft, shape of cross section: circular (0), horizontally-elongated elliptical (1), flat (2) (newly added).
4. Distal tibia–fibula articulation: relatively small (0), enlarged and extended proximally (1) (after Barnes, 2013, character 35).
5. Tibia, astragalar facet: not very tilted (= nearly perpendicular to shaft of bone) (0), tilted at least 60 degrees from shaft (1) (after Barnes, 2013, character 36).
6. Tibia: straight (0), medially twisted with its distal articular surface facing laterally (1) (after Inuzuka, 2000, 2005, character 3).
7. Phalanges: not flattened, distal ends only gently expanded medial and lateral side (0), flattened with greatly expanded distal ends (1) (after Clark, 1991, character 30).
8. Metacarpal 5, proximal end: not expanded (0), expanded laterally (1) (after Barnes, 2013, character 33).
