# Supplementary material for: The phylogeny of desmostylians revisited: proposal of new clades based on robust phylogenetic hypotheses
Source: PeerJ. 2019 Oct 17;7:e7430. doi: 10.7717/peerj.7430 (PMC6800978; doi:10.7717/peerj.7430)
Supplement: Table S1 [file peerj-07-7430-s002.pdf]

| | |
|---|---|
| Anthracobunids | 0 0 0 ? 0 0 0 0 0 1 0 0 0 1 0 0 0 0 0 0 0 0 0 0 0 0 0 0 0 0 0 0 0 3 0 - ? ? ? ? ? 0 ? 0 0 ? 0 |
| *Moeritherium* | 0 0 0 - - - 1 0 0 ? 0 0 0 ? 0 0 0 0 0 0 0 0 0 0 0 0 0 0 0 1 0 3 0 0 0 - 0 0 0 0 0 0 0 0 0 |
| *Pezosiren* | ? ? 0 0 0 0 0 0 ? ? 0 ? ? ? ? ? 0 ? ? 0 0 ? 1 ? 0 0 1 0 0 0 0 0 3 ? 0 ? - 0 0 0 0 0 0 0 1 0 |
| *Behemotops proteus* | 0 1 0 ? ? 1 0 0 0 2 0 0 0 1 1 1 0 0 0 0 0 0 1 0 0 0 1 0 0 1 0 ? 3 ? 1 ? ? ? ? 1 ? ? ? 0 ? ? |
| *Behemotops katsuiei* | 0 1 ? 0 ? 1 0 0 ? ? 0 0 0 ? 1 ? 0 0 0 0 0 0 0 1 0 0 1 0 0 ? ? ? ? ? ? ? ? ? ? ? ? 1 ? 0 ? ? ? |
| *Seuku emlongi* | 0 1 0 1 1 1 ? 0 ? ? 0 ? ? ? ? 1 0 ? ? 0 0 0 0 ? ? 0 1 ? 0 1 0 1 ? ? ? ? ? ? ? ? ? ? ? ? ? ? ? |
| *Archaeoparadoxia weltoni* | 0 1 0 0 1 ? 1 0 1 2 0 1 0 2 1 1 1 1 1 0 0 0 1 1 1 1 0 0 ? 1 0 1 ? 0 0 1 0 ? 1 1 1 ? 0 0 ? 0 |
| *Paleoparadoxia tabatai* | 0 1 0 0 1 1 1 0 2 2 1 1 1 2 1 1 1 1 1 0 0 0 0 1 1 1 0 0 0 1 0 1 3 1 0 1 0 1 1 1 1 0 0 0 0 ? |
| *Neoparadoxia repenningi* | ? 1 ? 0 1 1 1 0 ? ? 0 ? ? ? ? ? ? ? ? ? ? ? ? 1 ? ? ? ? ? 1 ? 1 ? ? ? ? ? ? ? ? ? ? ? ? ? ? |
| *Neoparadoxia cecilialina* | 0 1 0 0 1 1 1 1 2 2 0 ? 1 ? 1 ? 1 1 1 1 0 1 0 0 1 1 0 1 ? 1 0 1 3 1 1 1 0 1 1 1 1 0 0 0 0 0 |
| *Ashoroa laticosta* | 0 0 ? ? ? ? 0 0 0 ? 0 ? ? ? ? 1 1 1 1 1 0 0 0 ? 0 0 0 0 ? ? ? ? ? ? ? ? ? 1 ? 1 ? 1 ? 1 0 ? ? |
| *Cornwallius sookensis* | 1 0 0 0 1 1 1 1 - - 1 ? ? ? ? 1 1 1 1 1 1 0 ? 0 0 1 2 0 ? ? 1 ? 2 0 0 1 1 1 1 1 ? 0 0 0 1 1 |
| *Ounalashkastylus tomidai* | 1 0 0 ? 1 1 1 1 - - 1 - ? ? ? 1 1 1 ? 2 1 0 ? ? 1 ? 0 1 1 1 1 2 ? ? ? ? ? ? ? ? ? ? ? ? ? ? |
| *Desmostylus japonisus* | 1 0 0 0 1 1 1 0 - - 0 ? ? ? ? ? 1 1 0 2 1 1 ? ? 0 1 2 1 1 1 1 2 0 ? 0 ? ? 1 ? ? ? 0 ? ? 1 ? |
| *Desmostylus hesperus* | 1 0 1 0 1 1 1 1 - - 1 - 1 2 1 1 1 1 0 2 1 1 1 0 0 1 2 1 1 1 1 2 1 0 0 1 1 1 1 1 1 1 1 1 1 1 |
| *Desmostylus coalingensis* | 1 ? 1 0 ? 1 1 1 - - 1 - ? ? ? 1 1 1 0 2 1 1 1 ? 0 1 2 1 1 - 2 - 1 0 - ? ? ? 1 1 1 ? 1 ? ? ? |

| | | | | | | | | | | | | | | | | | | | | | | | | | | | | | | | | | | | | | | | | | | | | | | | | | | | | | | |
|---|---|---|---|---|---|---|---|---|---|---|---|---|---|---|---|---|---|---|---|---|---|---|---|---|---|---|---|---|---|---|---|---|---|---|---|---|---|---|---|---|---|---|---|---|---|---|---|---|---|---|---|---|---|---|
| 0 | ? | ? | 0 | 0 | 0 | 0 | 0 | 0 | 0 | 0 | 0 | ? | ? | 0 | 0 | ? | 0 | 0 | 0 | 0 | 0 | 0 | 0 | 0 | 0 | ? | 1 | ? | ? | ? | ? | ? | ? | ? | ? | ? | ? | ? | ? | ? | ? | ? | ? | ? | ? | ? | ? | ? | ? | ? | ? | ? | ? | ? |
| 0 | - | 0 | 0 | - | 0 | 0 | 0 | 0 | 0 | 0 | 0 | 0 | 0 | - | - | 0 | 0 | 0 | 0 | 0 | 0 | 0 | 0 | 1 | 0 | 0 | 0 | ? | 0 | 0 | 3 | 0 | 0 | 0 | - | 0 | 0 | 3 | 0 | 1 | ? | 0 | 0 | 0 | 0 | 0 | 0 | 0 | 0 | 0 | 0 | 0 | ? | ? |
| 0 | - | 0 | 1 | 0 | 0 | 1 | 0 | 1 | 0 | 0 | 1 | 1 | 1 | 0 | 0 | 0 | ? | 0 | ? | 0 | 0 | 0 | 0 | 0 | 0 | 0 | 1 | ? | 1 | 0 | 3 | 0 | 2 | 0 | - | 0 | 0 | 0 | 0 | 0 | ? | 0 | 0 | 0 | 0 | 0 | 0 | 0 | 0 | 0 | 0 | 0&1 | ? | ? |
| ? | ? | ? | ? | ? | ? | ? | ? | 0 | ? | ? | ? | ? | ? | ? | ? | ? | 0 | 0 | 0 | 0 | 0 | 1 | 0 | ? | 0 | 1 | ? | ? | ? | 0 | ? | ? | ? | ? | ? | 1 | ? | ? | ? | ? | ? | 1 | 0 | 1 | 1 | ? | 0 | 0 | ? | ? | ? | ? | ? | ? |
| ? | 1 | ? | 0 | 0 | ? | 0 | ? | 0 | 0 | 0 | ? | ? | ? | ? | ? | ? | 0 | 0 | ? | ? | 0 | ? | 0 | 1 | ? | ? | 1 | 0 | 0 | 0 | 0 | 0 | ? | ? | ? | 1 | 0 | 3 | ? | ? | ? | ? | ? | ? | ? | ? | ? | ? | 0 | 0 | 0 | 0 | 1 | ? |
| ? | ? | ? | ? | ? | ? | ? | ? | ? | ? | ? | ? | ? | ? | ? | ? | ? | 0 | ? | 0 | 0 | 0 | 1 | 0 | 0 | 0 | ? | ? | ? | ? | ? | ? | ? | ? | ? | ? | ? | ? | ? | ? | ? | ? | ? | ? | ? | ? | ? | ? | ? | ? | ? | ? | ? | ? | ? |
| 1 | 0 | 1 | 0 | 0 | 0 | 1 | 0 | 0 | ? | 0 | 1 | 0 | 0 | 0 | 0 | 0 | 0 | 0 | 0 | 0 | 1 | 1 | 0 | 1 | 1 | 1 | ? | ? | 0 | 0 | ? | 0 | ? | ? | 0 | 1 | 0 | 0 | 1 | 0 | 0 | 1 | 1 | 0 | 1 | 1 | 1 | 0 | 0 | 0 | 0 | 0 | 0 | 0 |
| 0 | ? | 1 | 0 | 1 | 0 | 1 | 0 | 0 | 0 | 0 | 1 | 1 | 0 | 0 | 0 | 1 | 0 | 0 | 1 | 0 | 1 | 1 | 0 | 1 | 1 | 1 | 1 | 1 | 0 | 0 | 1 | 1 | 0 | 0 | 0 | 1 | 0 | 1 | 1 | 2 | 0 | 1 | 1 | 0 | 1 | 1 | 1 | 1 | 0 | 0 | 0 | 0 | 0 | 1 |
| ? | ? | ? | ? | ? | ? | ? | ? | ? | ? | ? | ? | ? | ? | ? | ? | ? | 0 | 1 | 0 | 1 | 1 | 1 | 0 | 1 | 1 | 1 | 1 | ? | 0 | 0 | 2 | 1 | 1 | 0 | 1 | 1 | 0 | 2 | 1 | 0 | 1 | ? | ? | ? | 1 | ? | 1 | 1 | 0 | 1 | 1 | 0 | 1 | 1 |
| 0 | 0 | 1 | 0 | 0 | 1 | 1 | 0 | 0 | 0 | 0 | 1 | 1 | 1 | 1 | 1 | 1 | 0 | 1 | 0 | 0 | 1 | 1 | 0 | 1 | 1 | 1 | ? | ? | 0 | 0 | 1 | 1 | 0 | 1 | 1 | 1 | 0 | 3 | 0 | 0 | 1 | 1 | 1 | 0 | 1 | 1 | 1 | 1 | 1 | 0 | 1 | 0 | 0 | 1 |
| ? | ? | 1 | 1 | 0 | 0 | 0 | 0 | ? | 0 | 0 | ? | ? | ? | 1 | 0 | ? | 0 | 0 | 0 | ? | ? | ? | ? | ? | ? | ? | 2 | 1 | 1 | 1 | 0 | 0 | 0 | ? | ? | 1 | 1 | 0 | 0 | 2 | 0 | 1 | 1 | 0 | 0 | 1 | 0 | 0 | 0 | 0 | ? | 1 | ? | ? |
| 1 | 1 | 1 | 1 | 0 | 0 | 0 | 1 | 1 | 0 | 0 | 0 | 1 | 1 | 1 | 0 | 0 | 0 | 0 | 0 | 0 | 0 | 1 | 0 | 1 | - | ? | ? | ? | ? | ? | ? | ? | ? | ? | ? | ? | ? | ? | ? | ? | ? | ? | ? | ? | ? | ? | ? | ? | ? | ? | ? | ? | ? | ? |
| ? | ? | ? | ? | ? | ? | ? | ? | ? | ? | ? | ? | ? | ? | ? | ? | ? | 0 | 0 | 0 | 0 | 0 | 1 | 0 | 1 | - | ? | ? | ? | ? | ? | ? | ? | ? | ? | ? | ? | ? | ? | ? | ? | ? | ? | ? | ? | ? | ? | ? | ? | ? | ? | ? | ? | ? | ? |
| ? | ? | 1 | 1 | 1 | 1 | 1 | 1 | 0 | 1 | 1 | 0 | 0 | 0 | ? | ? | ? | ? | ? | ? | 0 | 0 | 2 | 0 | 1 | - | ? | ? | ? | ? | ? | ? | ? | ? | ? | ? | ? | ? | ? | ? | ? | ? | ? | ? | ? | ? | ? | ? | ? | ? | ? | ? | ? | ? | ? |
| 0 | 0 | 1 | 1 | 1 | 1 | 0 | 0 | 1 | 0 | 0 | 1 | 0 | 0 | 1 | 1 | 0 | 1 | 0 | 0 | 1 | 0 | 2 | 1 | 1 | - | 1 | 3 | 3 | 0 | 0 | 0 | 0 | 2 | 0 | 1 | 1 | 1 | 2 | 0 | 0 | 1 | 0 | 0 | 1 | 0 | 2 | 0 | 1 | 0 | 0 | 1 | 1 | 0 | 1 |
| ? | 1 | ? | 1 | 1 | 1 | 0 | 0 | 1 | ? | ? | ? | ? | ? | ? | 0 | 0 | 1 | 0 | 0 | 0 | 0 | 0 | 1 | ? | - | ? | ? | ? | ? | ? | ? | ? | ? | ? | ? | ? | ? | ? | ? | ? | ? | ? | ? | ? | ? | ? | ? | ? | ? | ? | ? | ? | ? | ? |

| | | | | | | |
|---|---|---|---|---|---|---|
| ? | ? | ? | ? | ? | 0 | ? |
| ? | 0 | ? | ? | 0 | ? | ? |
| 0 | ? | ? | 0 | 0 | 1 | ? |
| ? | ? | ? | ? | ? | 1 | ? |
| 1 | 1 | ? | 0 | 1 | 0 | 0 |
| ? | ? | ? | ? | ? | ? | ? |
| ? | ? | ? | ? | ? | 1 | 0 |
| 0 | 2 | 0 | 0 | 1 | 1 | 1 |
| 0 | 2 | 1 | 1 | 1 | 1 | 1 |
| 0 | 2 | 1 | 1 | 1 | 1 | 1 |
| 1 | 1 | ? | ? | ? | ? | ? |
| 0 | 1 | ? | ? | 1 | ? | ? |
| ? | ? | ? | ? | ? | ? | ? |
| ? | ? | ? | ? | ? | ? | ? |
| 1 | 1 | ? | 0 | 1 | 1 | 0 |
| ? | ? | ? | ? | ? | ? | ? |
