# Supplementary figures and images for: The phylogeny of desmostylians revisited: proposal of new clades based on robust phylogenetic hypotheses
Source: PeerJ. 2019 Oct 17;7:e7430. doi: 10.7717/peerj.7430 (PMC6800978; doi:10.7717/peerj.7430)

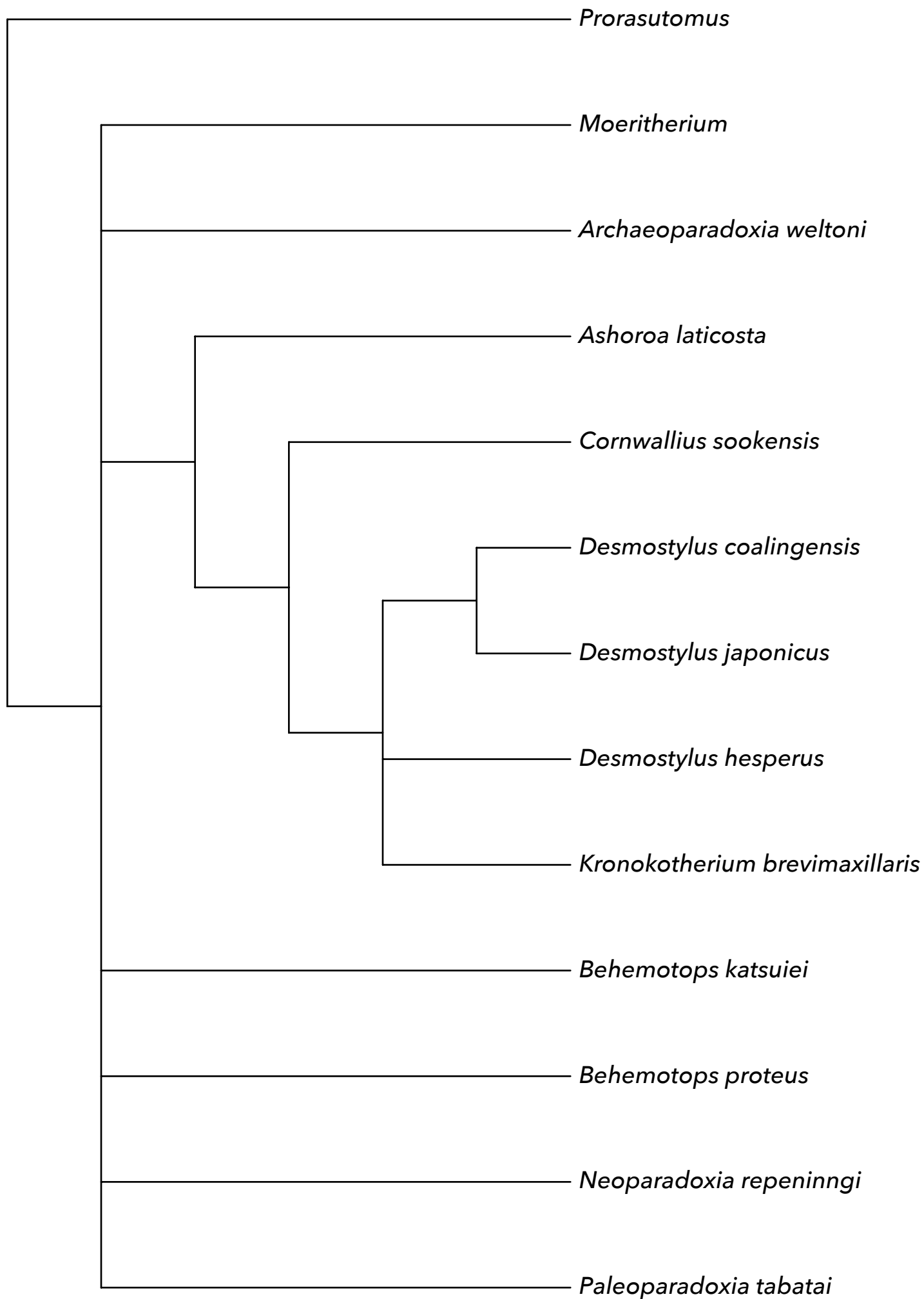

Supplement: Figure S1 [file peerj-07-7430-s003.pdf]

### Strict consensus tree

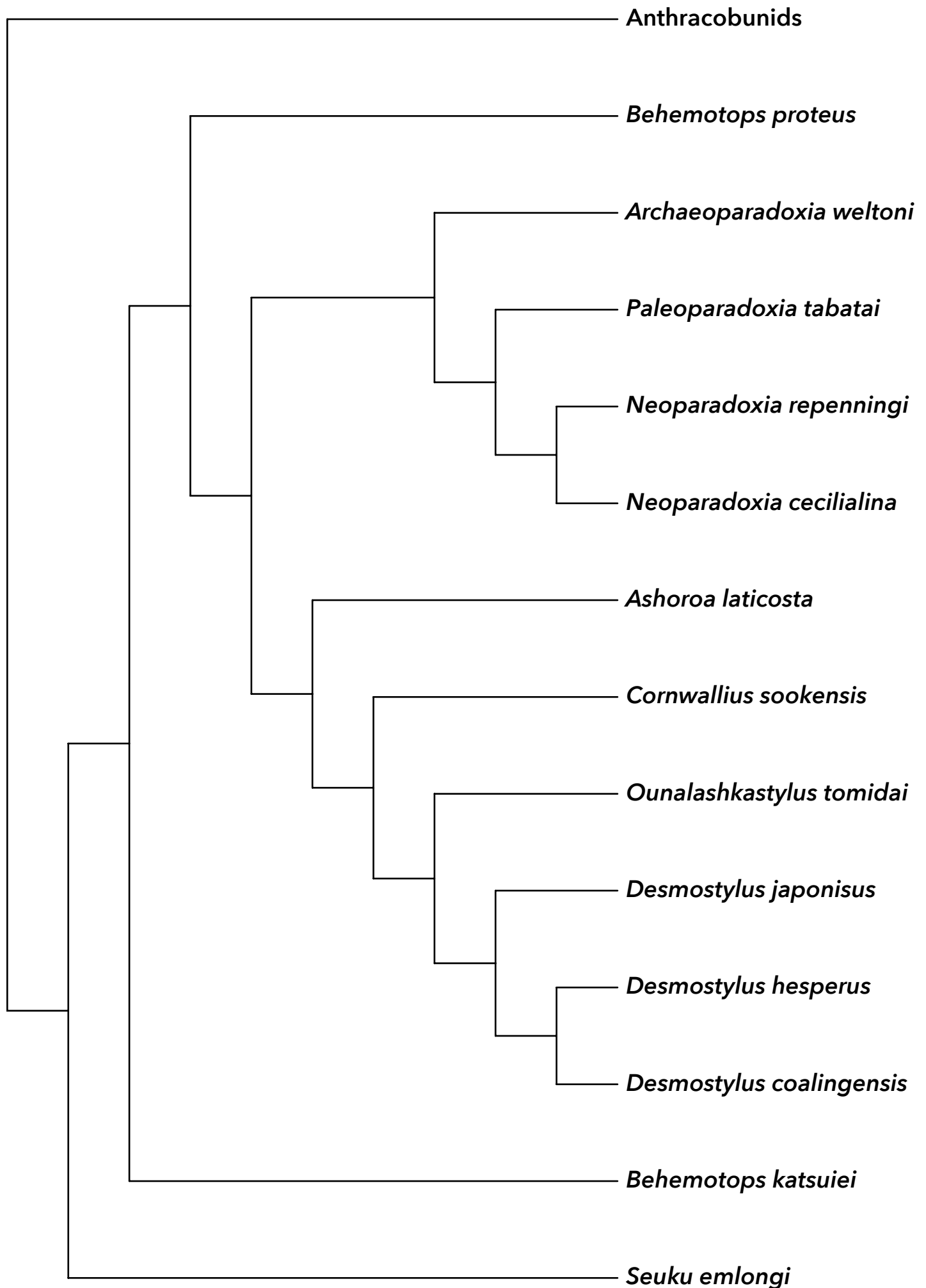

Supplement: Figure S2 [file peerj-07-7430-s004.pdf]

## Bootstrap consensus tree

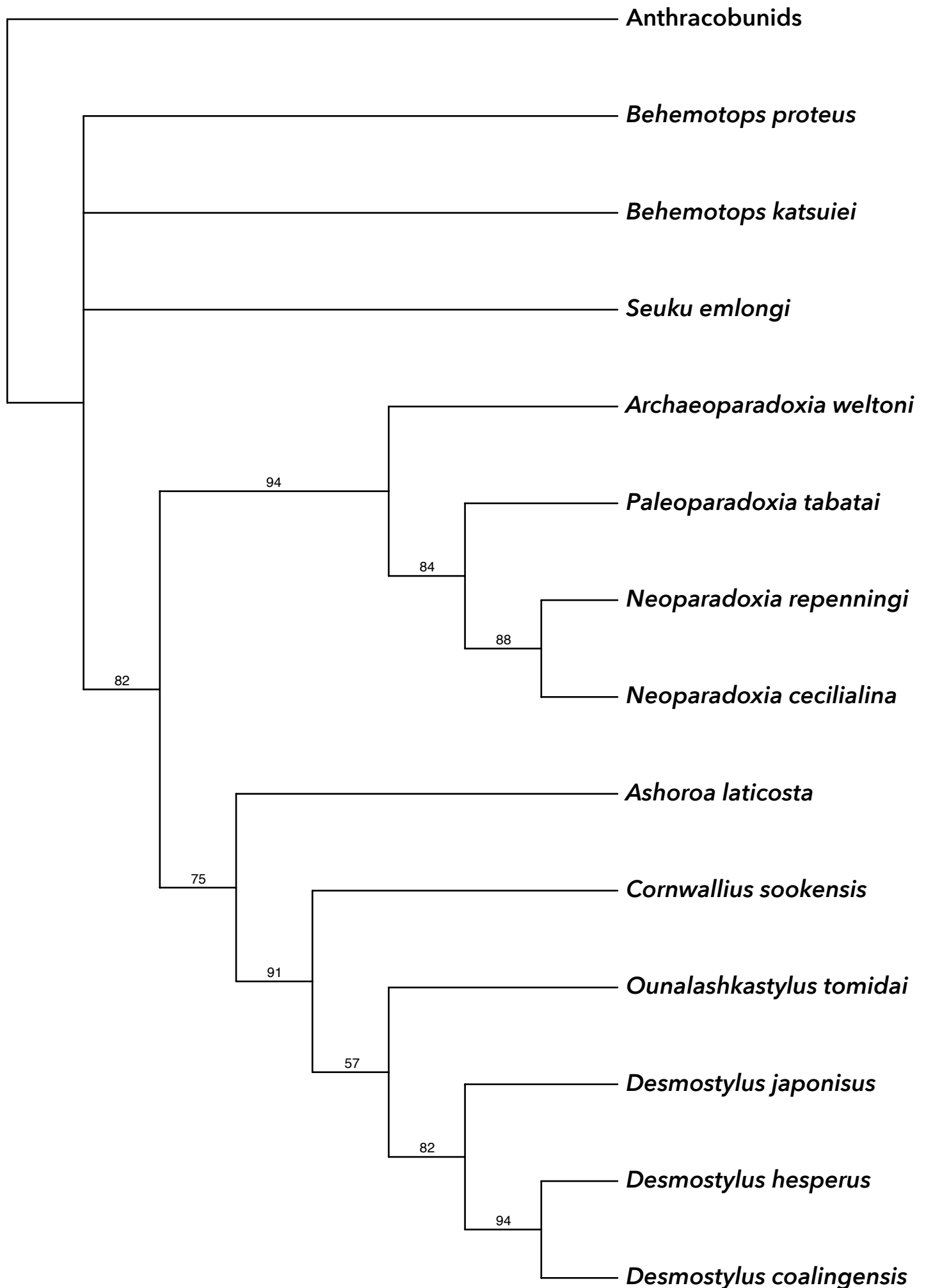

Supplement: Figure S3 — L = 163, CI = 0.699, RI = 0.688, RC = 0.481, HI = 0.301, G-fit = − 63.350 [file peerj-07-7430-s005.pdf]

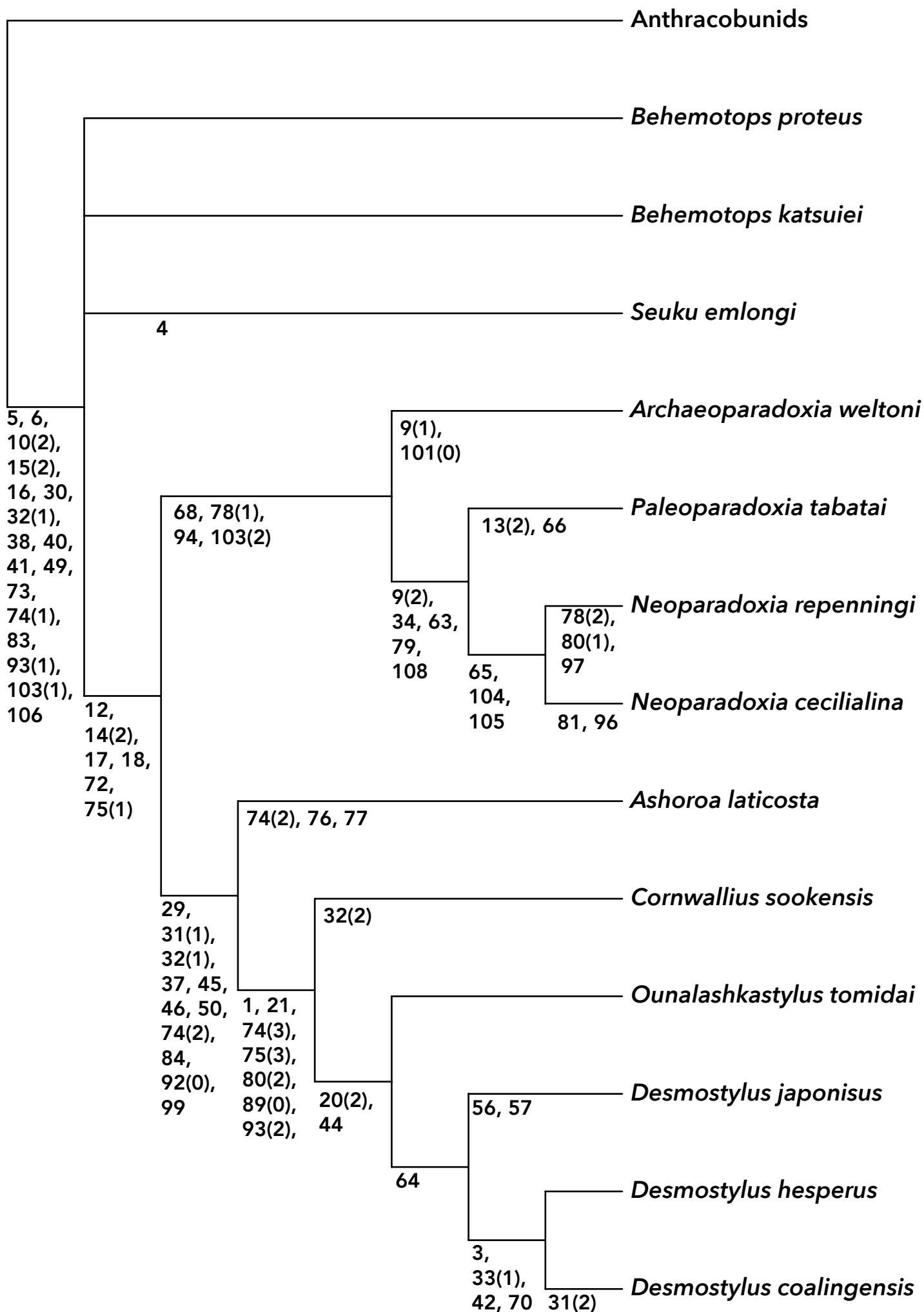

Supplement: Figure S4 [file peerj-07-7430-s006.pdf]

Strict consensus tree

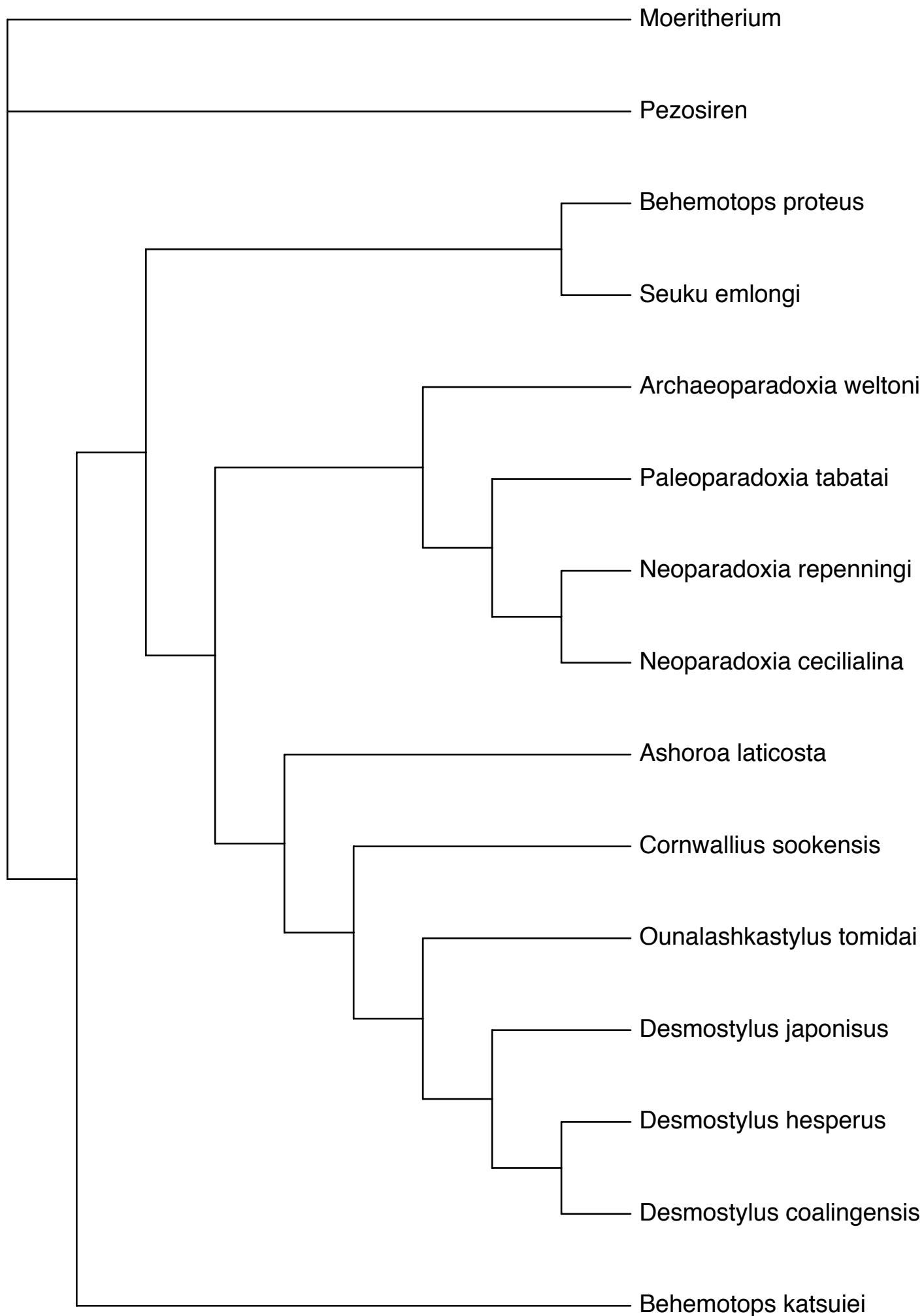

Supplement: Figure S5 [file peerj-07-7430-s007.pdf]

Bootstrap consensus tree

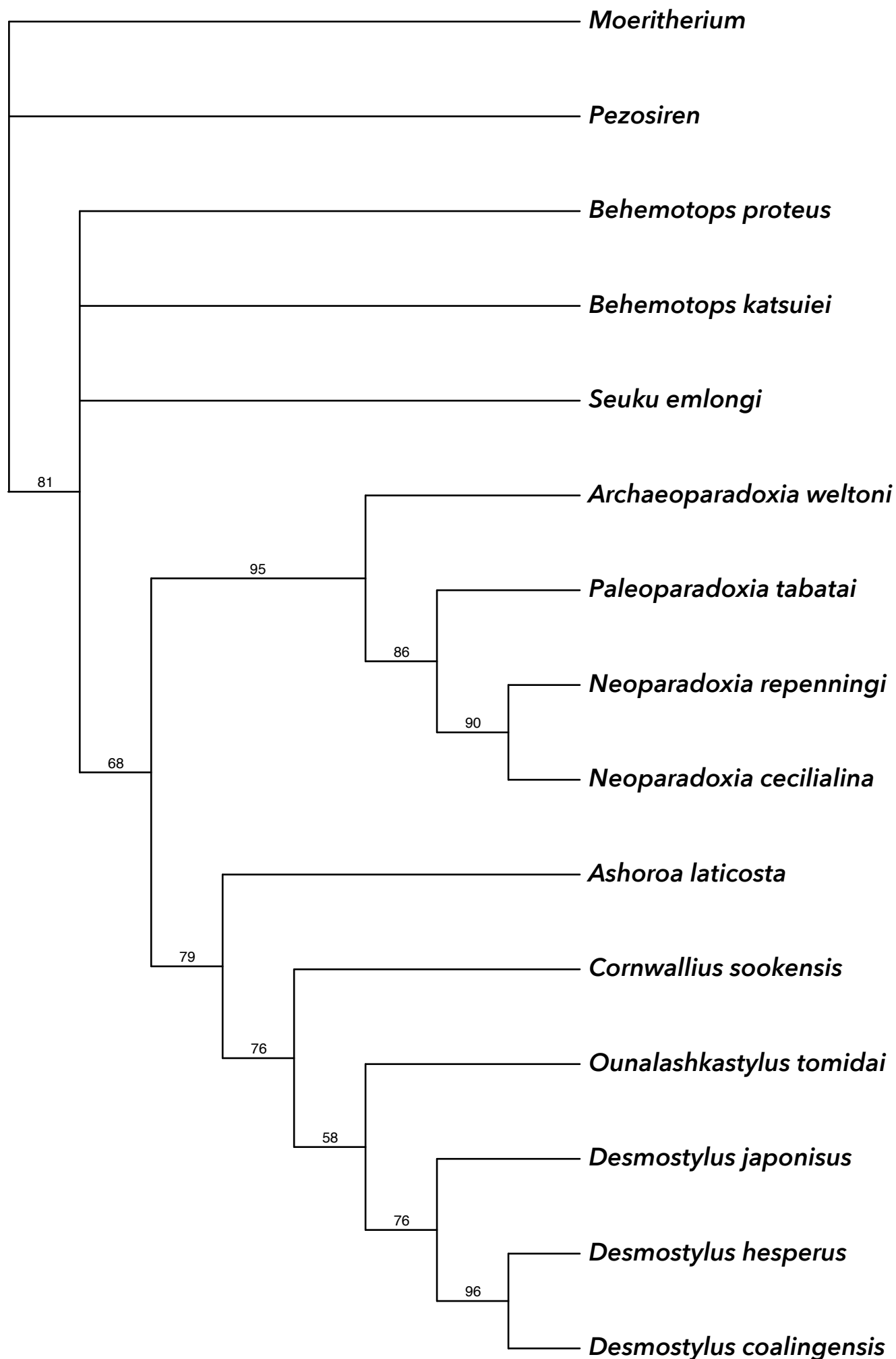

Supplement: Figure S6 — L = 190, CI = 0.663, RI = 0.663, RC = 0.440, HI = 0.337, G-fit = − 71.600 [file peerj-07-7430-s008.pdf]

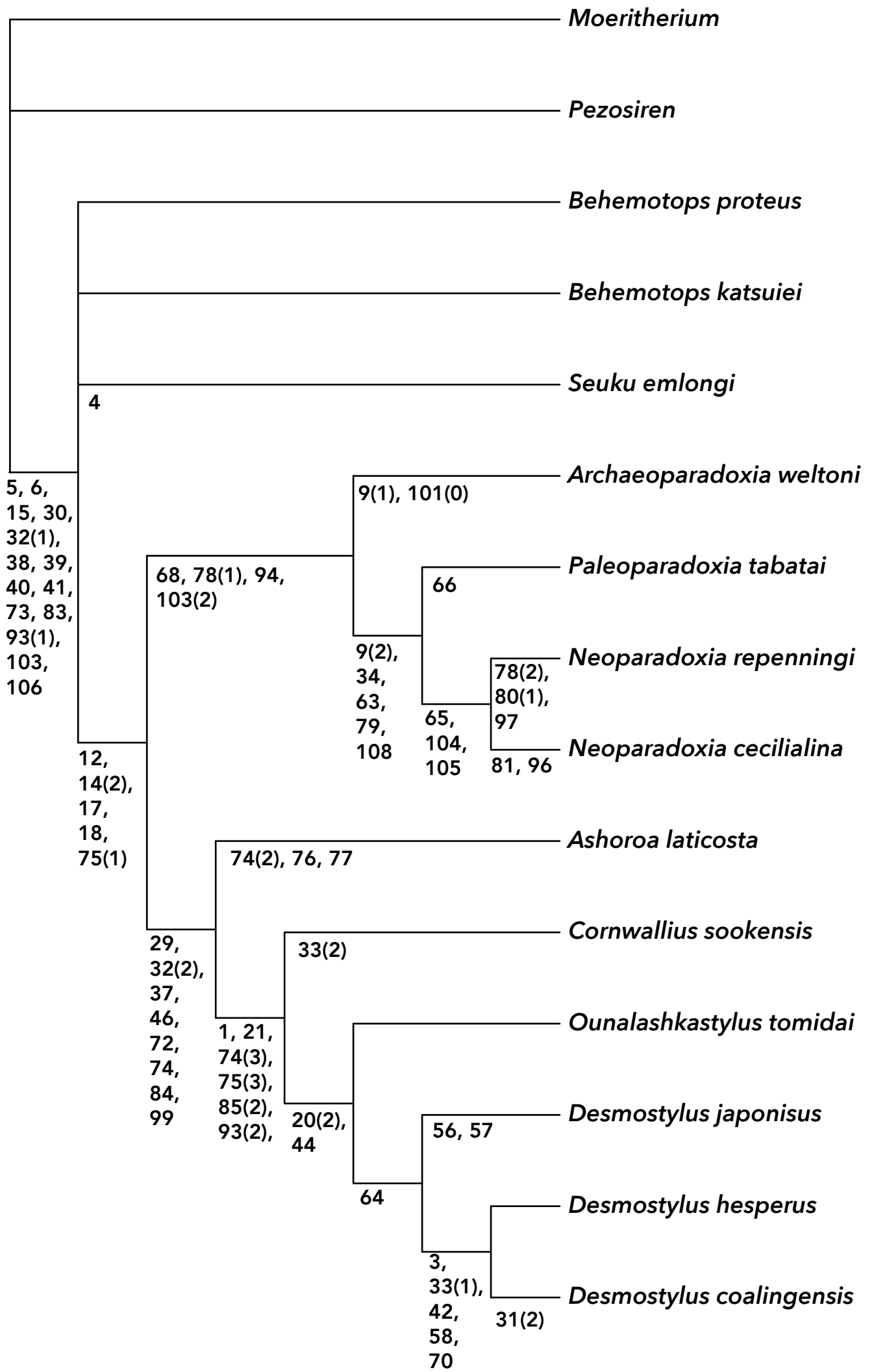

Supplement: Figure S7 [file peerj-07-7430-s009.pdf]

Strict consensus tree

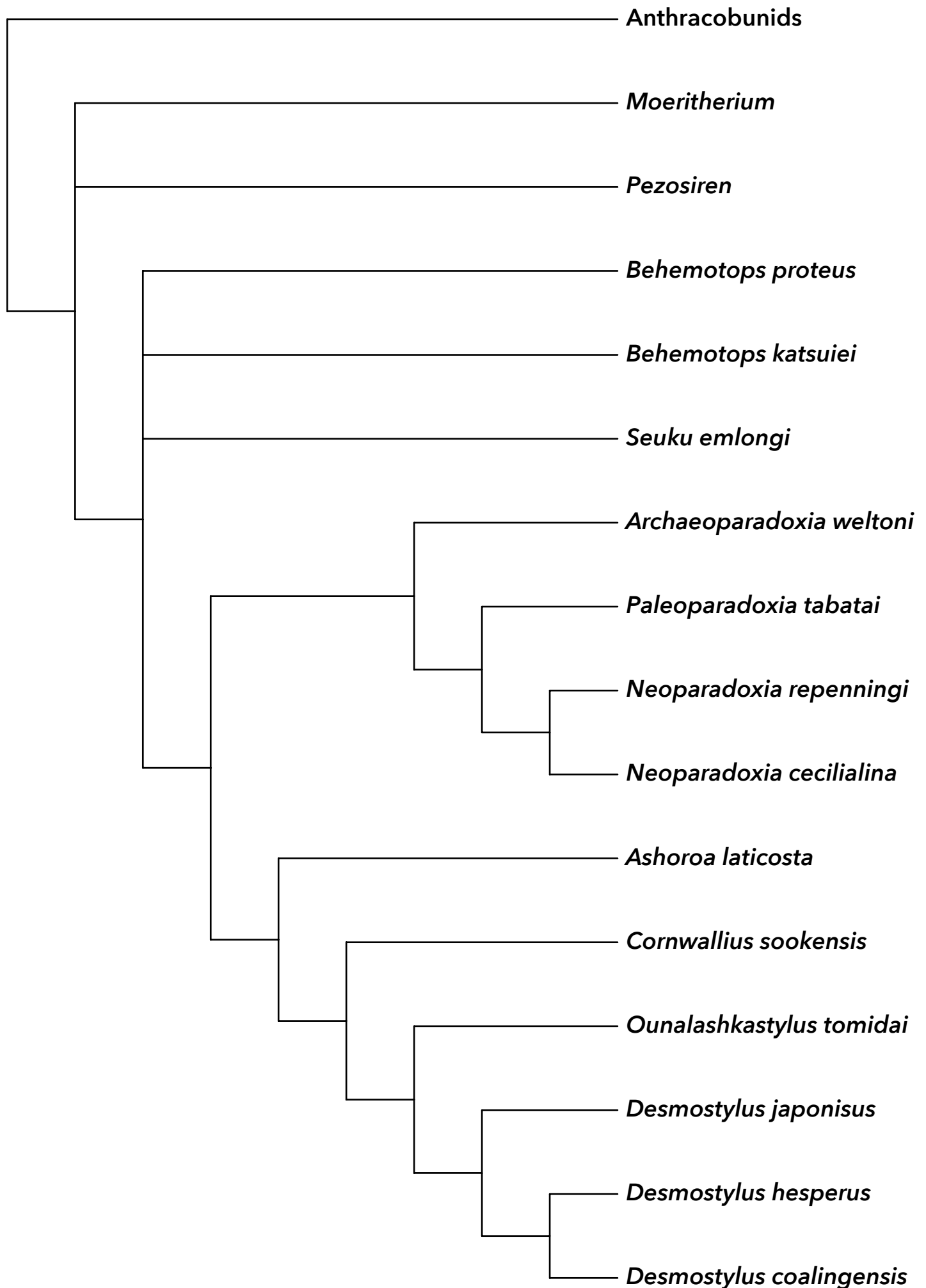

Supplement: Figure S8 [file peerj-07-7430-s010.pdf]

## Bootstrap consensus tree

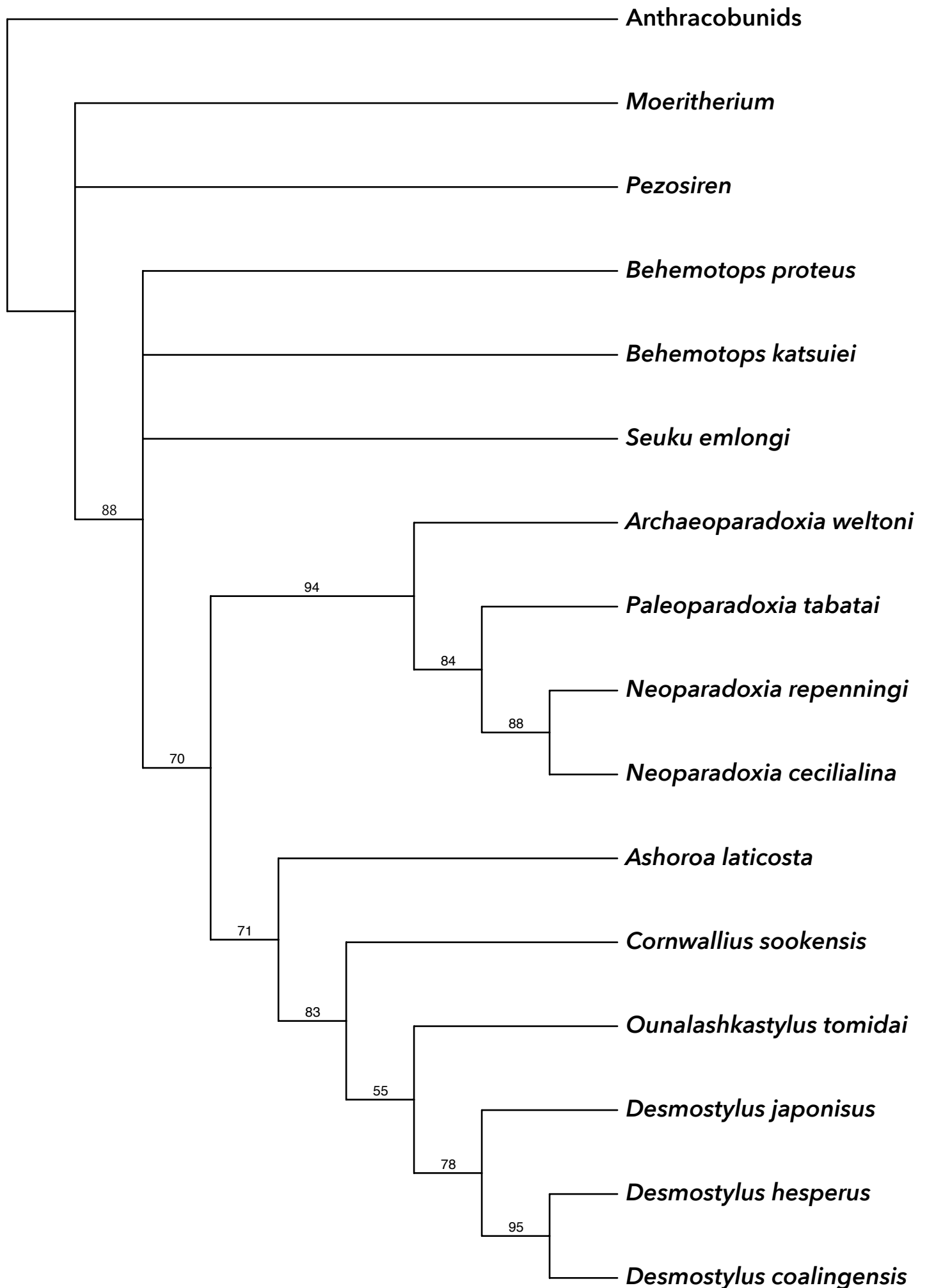

Supplement: Figure S9 — L = 194, CI = 0.655, RI = 0.679, RC = 0.445, HI = 0.345, G-fit = − 77.100 [file peerj-07-7430-s011.pdf]

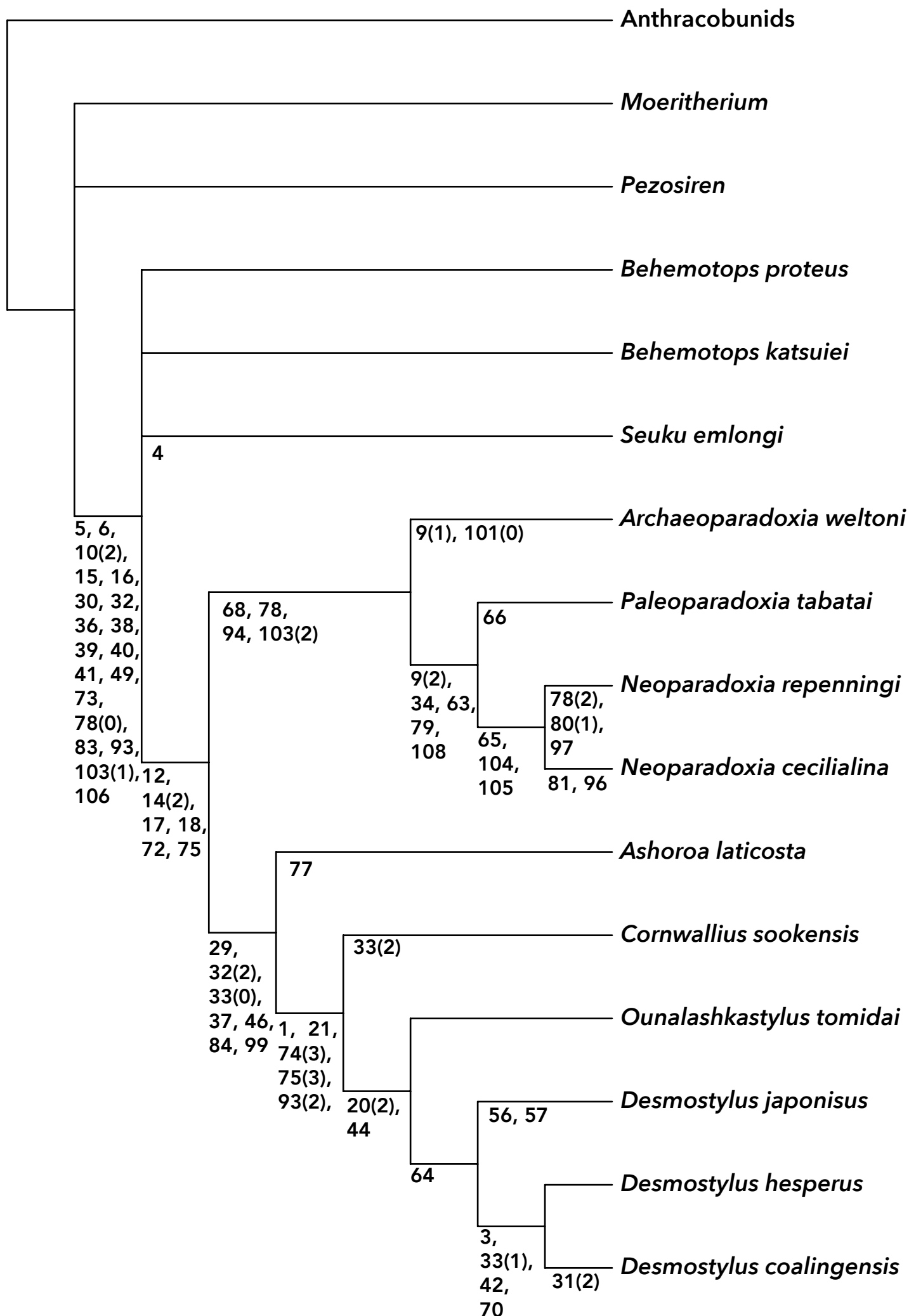

Supplement: Figure S10 [file peerj-07-7430-s012.pdf]
